# Multiclonality of ER expression in DCIS – Implications for clinical practice and future research

**DOI:** 10.18632/oncotarget.28450

**Published:** 2023-07-20

**Authors:** Mangesh A. Thorat

**Keywords:** ductal carcinoma *in situ* (DCIS), invasive breast cancer, ER, recurrence, clonality

**Figure 1:**
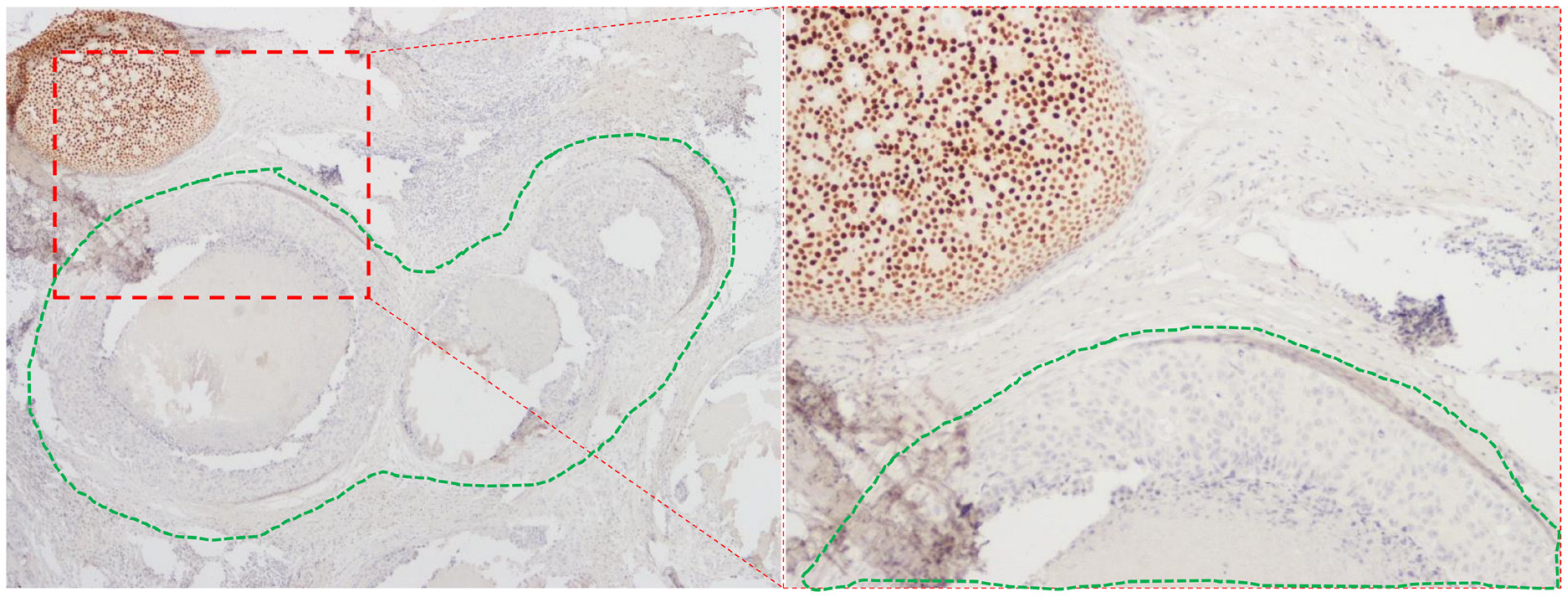
Multi-clonal ER expression in DCIS. Right hand panel (Magnification 20×) shows an area within the left-hand panel (Magnification 10×) containing adjacent CIS ducts with and without (encircled in green dotted line) ER expression.

Estrogen receptor (ER) expression is not routinely evaluated in DCIS, perhaps because the prognostic role of ER in DCIS was unclear until we showed in the UK/ANZ DCIS trial that lack of ER expression in DCIS was associated with a greater than 3-fold risk of ipsilateral recurrence [1]. This is the largest case-control study nested in a DCIS randomized trial and eliminated treatment allocation bias as well as treatment-related confounding through a meticulous study design. This is also the first ever study to show that ER expression in DCIS is multi-clonal with very important clinical and research implications [1]. During the study, I observed that a small proportion of otherwise ER-positive DCIS (Allred score of 3 or more on whole sections) also contained carcinoma *in situ* (CIS) duct/s that completely lacked ER expression (Figure 1). This admixture of clearly ER-positive and ER-negative ducts is not the same as heterogeneity in ER expression and I labelled such DCIS cases as multi-clonal DCIS even if just one CIS duct in the entire section lacked ER expression. Eighty-nine percent of ER-positive cases were true ER-positive (uni-clonal expression) and ER expression was multi-clonal in the remaining 11%. In such ER multi-clonal DCIS, two possibilities exist. The first possibility is that the ER-positive clone is the dominant clone that determines the outcome. In this case, the current method of labelling such DCIS as ER-positive would be valid, irrespective of whether ER expression is uni-clonal or multi-clonal. The second possibility is that the ER-negative clone is the dominant clone and therefore such DCIS will behave like ER-negative DCIS and ER-multi-clonal DCIS should really be classified as ER-negative as proposed in our “clonal method”. Indeed, we observed that outcomes in ER-multi-clonal DCIS are exactly like outcomes in ER-Negative DCIS (see Figure 1 of Thorat et al. [1]). Ipsilateral breast event (IBE) risk was higher in ER-multi-clonal [Matched Odds Ratio (mOR) 3.23; 95% Confidence Interval (CI), 1.48–7.04; *P* = 0.0033] and ER-negative DCIS (mOR 3.36; 95% CI, 2.04–5.51; *P* < 0.0001) as compared with uni-clonal ER-positive DCIS and the effect sizes were similar (mOR 3.23 vs. 3.36). The clonal method was more informative (Δχ^2^ 9.47) than the current standard method of assessing ER expression and when ER status was ascertained by the clonal method, ER-negative DCIS had a five-fold higher risk of *in situ* IBE (DCIS-IBE) (mOR 4.99; 95% CI, 2.66–9.36; *P* < 0.0001), but the risk of invasive IBE (I-IBE) was not significantly elevated (mOR 1.72; 95% CI, 0.84–3.53; *P* = 0.14), *P*_heterogeneity_ = 0.03. ER was an independent predictor in multivariate analyses (mOR 2.66 95% CI, 1.53–4.61; *P* = 0.0005) and significantly (Δχ^2^ (1d.f.) 13.28; *P* = 0.0003) improved the multivariate model of important clinicopathological variables. PgR was not significantly associated with the recurrence risk in ER-positive DCIS and did not add to the prognostic information already provided by ER. In summary, ER is a strong and independent prognostic biomarker in DCIS and our novel clonal method is a more accurate method to assess ER status in DCIS.

We have discussed in detail the clinical implications of ER in avoiding overtreatment and undertreatment in DCIS [1]. ER-negative DCIS has a high recurrence rate even after receiving radiotherapy [1, 2] and therefore for large lesions such as those requiring advanced oncoplastic procedure with complex localization (e.g., bracketing), mastectomy [3] may be a preferred option that also avoids radiotherapy. With excellent immediate reconstruction options available now, such local treatment option may also be preferred by patients following a thorough informed discussion. On the other hand, low or intermediate grade DCIS with small tumor size is often overtreated with radiotherapy. If DCIS is uni-clonal ER-positive, such patients could easily avoid overtreatment in the form of radiotherapy and potentially opt for endocrine therapy that also reduces contralateral breast cancer risk [4–6].

Multiclonality and clonal evolution in cancers have regained importance after high-throughput genomic studies demonstrated their importance [7]. In DCIS, the clones with different temporal and/or spatial evolutionary path are contained within their individual geographically separated duct silos. This allowed us to observe such multiclonality based on morphological and protein expression changes detected by simple assays. The geographic separation among different clones is lost as the clones get admixed when DCIS progresses to invasive breast cancer. Therefore, DCIS represents an easier model [8, 9] to investigate differences in the biological behavior of different clones as well as therapeutic resistance and clonal evolution under therapeutic selection pressure [10].

Lastly, our study also proves the importance of clinical observation. It is possible that I was the first surgeon to notice the differential ER expression in CIS ducts under the microscope, but I am certain that many pathologists would have observed it before. It could also be argued that I was fortunate to have access to this large cohort of pathology material from a mature randomized trial with outcome data so that I could systematically investigate the importance of this observation. However, it is worth noting that we assembled this large cohort with the intent of investigating DCIS biomarkers based on simple, readily available assay methods that could be easily implemented anywhere in the world. Therefore, it is reasonable to rule out serendipity. This work provides a message for any researcher, and especially young budding researchers, clinicians and medical students. Multi-million-dollar research projects using advanced technology are not the only ones to produce scientifically or clinically meaningful results. Simple clinical observations made with an open mind and inquisitiveness can also provide important insights, provided such observations are systematically investigated by applying robust scientific methodology. Important “bedside” discoveries are still possible!
